# Optimizing HIV case identification among children and understanding remaining gaps in pediatric HIV testing in Kinshasa, DRC

**DOI:** 10.1186/s12887-023-04485-1

**Published:** 2024-01-04

**Authors:** Dominique Ingala, Winnie Bakebua, Fideline Banzadio, Dieudonne Tshishi, Aime Loando, Aimé Mboyo, Michelle M. Gill

**Affiliations:** 1 Elizabeth Glaser Pediatric AIDS Foundation, Kinshasa, Democratic Republic of Congo; 2 National AIDS Control Program, Kinshasa, Democratic Republic of Congo; 3 Elizabeth Glaser Pediatric AIDS Foundation, Washington, DC USA

**Keywords:** The Democratic Republic of the Congo, HIV testing, Children

## Abstract

**Background:**

It is critical to identify children living with HIV and initiate lifesaving antiretroviral treatment (ART) early. The Pediatric Accelerated Case Finding Effort focused on contact elicitation and HIV testing of ART clients’ biological children. We describe HIV testing and seropositivity rates following the initiative and gaps along the index testing cascade to inform pediatric HIV case finding optimization.

**Methods:**

This mixed-methods study involved collecting monthly data on index testing outcomes, including elicitation (identifying biological children < 15 years), HIV testing and linkage to treatment from March 2020 to July 2021 in 35 facilities in Kinshasa. Data were summarized and presented for the first month (as a baseline proxy) and the entire study period. Qualitative data were collected from 14 healthcare workers participating in in-depth interviews and 33 community health workers in four focus group discussions. Audio recordings were transcribed and translated from Lingala or French into English and coded using MAXQDA software. Data were thematically analyzed according pediatric case finding barriers and strategies.

**Results:**

At baseline (March 2020), among 3337 eligible female index clients, 1634 (49.0%) underwent elicitation to identify children with unknown HIV status. By July 2021, all eligible clients (*n* = 11,734) had contacts identified. Of the contacts, 9871/11,848 (83.3%) were HIV-tested. Of contacts tested, 662 (6.7%) were diagnosed as HIV-positive, with 535 (80.8%) age 5–14 years; 99.5% initiated treatment. Providers attributed gaps in HIV testing primarily to testing refusals for children due to non-disclosure among parents and logistical or financial obstacles to transportation for tracing. COVID-19 movement restrictions and exposure fears also limited provider interactions for testing. Provider-implemented strategies included transport reimbursement, extensive counseling and alternative approaches to child testing for parents in sero-discordant relationships.

**Conclusions:**

Following intensified efforts around pediatric case finding, we found a high HIV positivity yield of 6.7% among previously undiagnosed children, with 81% of them aged ≥5 years. While elicitation improved over time, contact tracing for HIV testing remained the largest gap, missing opportunities to reach 17% of undiagnosed children. Ensuring adequate resources for tracing and HIV testing and supporting disclosure among couples, while emphasizing elicitation of ART clients’ biological children can help to optimize pediatric case finding.

**Supplementary Information:**

The online version contains supplementary material available at 10.1186/s12887-023-04485-1.

## Background

Through 2021, only 52% of children living with HIV (CLHIV) globally were receiving antiretroviral treatment (ART), compared to 76% of adults [1]. While improvement has been seen for adults, compared to previous years, children aged 0–14 years continued to lag behind, thereby accentuating disparities between adults and children. Regional disparities were also greater, with lower ART coverage of children aged 0–14 years in western and central Africa (35%) compared to 56% in eastern and southern Africa [1]. In the Democratic Republic of the Congo (DRC), only 38% of CLHIV aged 0–14 years received ART compared to 88% of adults [2].

Although reaching and diagnosing CLHIV is complex, the global community has the evidence, tools and know-how to accelerate pediatric HIV case finding [3]. However, pediatric HIV case finding has not been prioritized [4]. Despite efforts to close this gap, CLHIV aged 5–14 years are likely missed more frequently than younger children, once they age-out of under five-focused health services. Expansion of case finding strategies and targeted approaches are needed in this age group [5].

A critical case finding approach particularly for older children who may have been missed by early infant diagnosis programs is index testing [6]. Index testing is a voluntary process that is undertaken by a healthcare worker (HCW) or community health worker (CHW) soliciting index clients to list all of their children and sexual partners in the past 12 months. This is followed by subsequent contact tracing and HIV testing of children and sexual contacts undiagnosed with HIV. Testing is conducted in a health facility or in the household/community, and those testing HIV-positive should then be linked to ART [6]. These steps are referred to in this paper as the pediatric index testing ‘cascade’: child contact elicitation (identifying contacts and determining their eligibility for HIV testing), contact tracing and HIV testing, and linkage to treatment if diagnosed as HIV-positive.

Index testing is subject to several barriers that can occur at different levels throughout the process. They may be individual, family, or health system challenges. These barriers include fear of stigma from friends and providers, fear of consequences of a positive test, including intimate partner violence, prioritization of HIV testing services (HTS) for other populations, transport constraints, HCW reluctance to provide HIV testing to children, and poor counseling approaches [3, 6, 7].

The Elizabeth Glaser Pediatric AIDS Foundation (EGPAF) introduced the pediatric-focused accelerated case finding effort (PACE) initiative in March 2020 to improve identification of children and adolescents < 15 years living with HIV. This study aimed to describe HIV testing and seropositivity rates following the PACE initiative and to contextualize the quantitative findings by interviewing facility and community providers on the process, the gaps and strategies to address those gaps along the pediatric index testing cascade.

## Methods

### Intervention description

The PACE initiative was implemented in 35 sites in Kinshasa in five health zones (HZ). Activities under the initiative targeted both HCW and CHW. The initiative included conducting an in-person two-day training for HCW and CHW on provider-initiated testing and counseling, the elicitation process, contact tracing, linkage to treatment, documentation, and safety and confidentiality issues around index testing. Other training topics included use of revised tools, such as the family tree form used for elicitation, allowing for better tracking of pediatric HIV testing, and a standard operating procedure (SOP) with step-by-step instructions on index testing and documentation. One HCW and two CHW were trained in each site; at high volume sites, where there are separate prevention of mother-to-child transmission (PMTCT) and HIV care and treatment clinics, two HCW received training. There were also short-term staff hired at the beginning of implementation to help ensure fidelity to pediatric case finding activities and monitor relevant provider activities. Finally, HZ management teams supervised case finding activities (visiting approximately one facility per week) and conducted weekly data reviews together with EGPAF. These activities were temporarily paused for about 3 months, from April to June 2020, as a result of lockdown measures in response to the COVID-19 pandemic. Activities resumed in July 2020 with the use of personal protection equipment and other barrier measures.

To conduct the index testing process, records of potentially eligible new and existing clients ≥18 years and receiving ART were extracted by HCW and CHW from the national HIV electronic database, TIER.Net (version 1.10.7). Records were exported to an Excel file and restricted to women of childbearing age. Information on clients’ contacts was only available from facility-based records. Since ART clients provide contact information at the time of enrollment in HIV care and treatment, and are contacted as part of routine service delivery, clients with missing or incomplete information would be invited to the facility or visited at home to obtain names and ages of eligible children, as applicable. Eligibility was defined as children and adolescents under 15 years of age with unknown HIV status whose mother was living with HIV. Unknown status was defined as no previous documentation of a confirmed positive or negative test result. Young children who were currently being breastfed were excluded from elicitation. The Excel files were used as a basic tracking document to reach index cases and proceed with elicitation. If there were already child contacts listed in the index client’s file, the relevant data would be abstracted, and providers would proceed directly to tracing the child/caregiver for HIV testing.

## Data sources and collection methods

### Clinic record collection

All information on the index testing process was captured using the family tree form and index case register. Data were collected monthly from March 1, 2020 to July 31, 2021 in the 35 intervention facilities. Data were entered into an Excel database and summarized descriptively across facilities and months. We present data from the entire 17-month period and from the first month of the initiative (March 1–31, 2020) as a proxy for baseline data. To minimize the risk of double counting, data clerks conducted weekly verification prior to reporting using unique client identification numbers.

### Interviews and focus group discussions

Qualitative data were collected in December 2020 from 19 of the 35 health facilities. We purposively selected four sites from each of the 5 HZ (though one had to be excluded due to ineligibility determined later) to help ensure a representative mix of health centers and hospitals and high (≥500 clients on ART) and low (< 500) client volumes in each HZ to the extent possible. Three hospitals and 16 health centers were included in this study component. FGD were conducted with CHW from all 19 sites; IDI were conducted with HCW in 11 sites.

For the study, all providers at study sites who received training on pediatric case finding under the PACE initiative, 1–2 HCW and two CHW per facility, were potentially eligible to participate in in-depth interviews (IDI) and focus group discussions (FGD), respectively. Providers also had to be providing HIV testing services, including index testing strategies involving children (0–14 years) at the time of recruitment. Of 18 HCW recruited, 14 participated in IDI. The reason given for non-participation was unavailability due to other commitments. Of 34 CHW recruited, 33 participated in four FGD (7–10 in each group); one CHW was consented, but did not present for the FGD.

Trained research assistants administered written informed consent in French for HCW and in French or Lingala (per participant preference) for CHW. All participants consented prior to data collection. Participants were first asked close-ended questions on demographics (age, gender) and professional history (years of service, HTS-related responsibilities). IDI and FGD were conducted in Lingala or French by one (IDI) or two (FGD) research assistants. As part of training, research assistants role-played and conducted practice sessions with non-participants, debriefing after each session and refining the interview guides to help ensure quality and consistency during data collection. They were also guided by an SOP with instructions for each question on the guides. Participants were asked about their perceptions of barriers along the pediatric index testing cascade and their strategies for addressing gaps in pediatric case finding implementation.

Audio recordings were simultaneously transcribed verbatim and meaningfully translated into English by a consultant with training and experience in qualitative research, fluency in Lingala, French, and English and strong familiarity with the program. Two other study team members, who are also fluent in all three languages, reviewed a subset of the audio recordings against the transcripts and reviewed all translated transcripts, which were then revised with the translator as needed. Data were coded and analyzed using MAXQDA (version 2020) using thematic analysis by participant group. A preliminary codebook was developed with deductive codes based on the qualitative guides and was refined along the way using an inductive approach based on the data. The final codebook and analysis were organized according to overarching themes (e.g., index testing gaps), each with a group of codes that correspond to each theme (e.g., elicitation step gaps). This paper focuses on the qualitative results that help to contextualize the quantitative findings. Major themes described below are the key factors contributing to gaps in pediatric case finding at each step along the cascade, the cross-cutting effect of COVID-19 on pediatric HTS and provider-identified strategies to help address those gaps. IDIs and FGDs explored additional themes, such as training and capacity strengthening under the PACE initiative.

### Ethical considerations

Data collected as part of this study was approved under two separate study protocols by The University of Kinshasa School of Public Health Institutional Review Board (IRB) and Advarra IRB in the United States.

## Results

### Pediatric index testing outcomes

In the first month of the PACE Initiative in March 2020, records of 4737 female index clients at study health facilities were reviewed. Of these, 3337 (70.4%) were women eligible for contact elicitation, defined as ≥1 biological child < 15 years with an unknown HIV status. Of the 3337 eligible index clients, 1634 (49.0%) underwent elicitation by a provider resulting in the identification of 2327 pediatric contacts eligible for HIV testing. Of the 2327 contacts, 1919 (82.5%) were tested at facility or community level; 408 (17.4%) pediatric contacts were not found for HIV testing or they were traced, but testing was declined. Of pediatric contacts tested, 112 (5.8%) tested positive for HIV. Nearly all (97.3%) of the contacts testing HIV-positive were linked to treatment; the remaining three children initiated ART after March 2020. An additional figure illustrates these results in more detail (see Additional file 1).

For the total study period, from March 2020 to July 2021, records of 13,176 female index clients ≥18 years on ART at study health facilities were reviewed to identify eligible pediatric contacts. All of these clients underwent elicitation of their biological child contacts. Of the 13,176, 11,734 (89%) were women living with HIV with at least one biological child under 15 years of age who had an unknown HIV status. Elicitation of the 11,734 eligible index clients resulted in the identification of 11,853 pediatric contacts. Among them, 11,848 were eligible for HIV testing; the five children ineligible for testing had already been diagnosed as HIV-positive; their status was not previously documented in clinic records. Of the pediatric contacts elicited, 2428 (20.5%) were 0–4 years, 4969 (41.9%) were 5–9 years, and 4451 (37.6%) were 10–14 years.

**Fig. 1:**
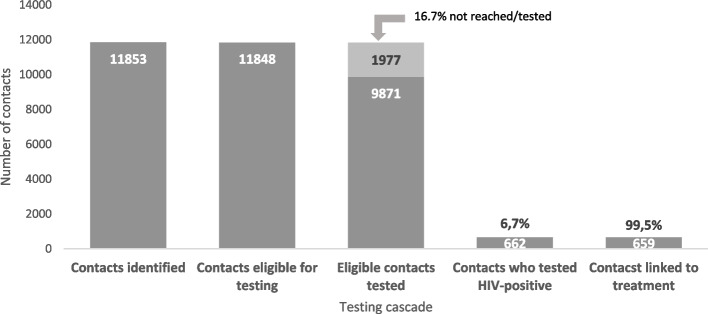
Pediatric HIV testing eligibility, testing and treatment cascade from March 2020 to July 2021

Of the 11,848 contacts, 9871 (83.3%) children were tested at facility or community level; 1977 (16.7%) pediatric contacts were not found for HIV testing or they were traced, but testing was declined (Fig. 1). Of pediatric contacts tested, 662 (6.7%) tested positive for HIV; 535 (80.8%) contacts were 5–14 years of age. Nearly all (99.5%) of the contacts testing HIV-positive were linked to treatment; the remaining three children initiated ART later (after July 2021) following further counseling with their caregivers.

### FGD and IDI participant characteristics

The median age of participants participating in qualitative data collection was 45.0 and 41.5 years, respectively for CHW and HCW; the majority were female (Table 1). Among the HCW, the majority interviewed were nurses. There was a median of three CHW per site and a median of five HCW per site providing HIV services, though staffing volumes varied greatly across sites.

**Table 1:** Demographics of community and healthcare workers who participated in the qualitative study

Group	CHW (*n* = 33)	HCW (*n* = 14)
Age, median (IQR)	45.0 (39.3, 53.0)	41.5 (36.5, 44.5)
Gender, female, n (%)	25 (73.5)	9 (64.3)
**Education**		
Primary	2 (5.9)	–
Secondary	26 (76.5)	–
Higher	6 (17.6)	–
**HCW Cadre**		
Nurse	–	9 (64.3)
Doctor	–	2 (14.3)
Other	–	3 (21.4)
Time providing HIV services, median (IQR), in years	3.0 (4.5, 7.8)	6.5 (3.5, 8.0)
Number of staff providing HIV services, median (IQR), range	3.0 (2.0, 3.0), 1–10	5.0 (4.0, 13.5), 1–21

### Gaps along the index testing ‘cascade’

The primary gaps identified through qualitative data collection were identifying children of ART clients through line-listing and reaching children for HIV testing.

### Contact elicitation

The majority of participants described identifying child contacts at index cases’ initial appointments and about half reported that this was done for new clients or as part of subsequent clinic visits for returning clients if there was not an existing contact list. HCWs and CHWs acknowledged the challenge of incomplete lists of biological children. One commonly reported reason was parental resistance. Not all mothers agreed to divulge that they had children or that they were married or had a partner, but some parents reportedly resisted indirectly, as perceived by the respondents, by not giving the exact or complete number of children or offering conflicting information. CHWs perceived mothers as providing excuses for why the child cannot be tested.

*“Of all these questions, out of ten [women], only two, for example, agree to say how many children they have and that they live with their children; the others will tell you: ‘ah, the children are traveling, we have separated them with their father, they went to this village, or to another province.’ We often encounter these kinds of cases.” (CHW).*

There were also programmatic changes and weaknesses that helped to explain elicitation gaps. Several HCWs and CHWs indicated that elicitation was not done previously because of a lack of will or promotion among implementing partners.

*“I do not actually think it was the workload because nowadays the workload is even bigger because the tasks are more complex now. I think it was simply a lack of awareness because we were not trained or briefed on the importance of getting index cases to list their contacts. But now that we have understood this, we are asking for more and more index cases to give us the names of their children.” (HCW).*

Several CHWs acknowledged the heavy burden on HCWs as a possible reason for inconsistent elicitation. Some HCWs reportedly did not appreciate the involvement of CHWs in the index testing process, especially because of the larger financial payment provided to the CHWs for conducting more of the community tracing (and thus requiring greater coverage of transportation costs). Negligence or forgetfulness was also mentioned a few times (mostly in HCWs’ descriptions), indicating that forms would be completed but missing the necessary information, particularly when forms are finalized after clients leave the clinic.

### Contact tracing and HIV testing

The majority of HCWs and a few CHWs described calling parents (existing clients) to schedule an appointment for children’s HIV testing. Appointments were also made during home visits for parents who did not have a telephone or those who did not desire to be called. Some parents brought their children along for HIV testing at the time of their own clinical visit to the health facility.

Fear of HIV status disclosure was overwhelmingly cited most frequently by both HCWs and CHWs to index testing overall. This negatively impacted provider ability to reach children for testing through home or clinic visits, particularly if index clients agreed and then changed their minds. Respondents explained that index clients’ fear was that their own HIV status would be disclosed to their HIV-negative partner or partner of unknown status (often husbands) or others they live with if they were to have their children tested for HIV.

*“But it’s more difficult for mothers who are afraid. A woman who hasn’t shared her HIV status with her partner is still living in fear. She is doing her best to protect her marriage and avoid being discovered that one of her children is HIV-positive…So the mother manages that situation while refusing to have her children tested because if her husband finds out, her home will be in danger.” (CHW).*

When asked to bring their children to the clinic for HIV testing, respondents reported that parents may refuse due to logistical or financial reasons. A few providers described caregivers continually deflecting, indicating they forgot to bring the children or that they will do it the next time they come to the clinic. However, when it is offered for someone to come to the home to address clinic-related challenges, respondents explained that the caregiver may refuse outright.

The most commonly mentioned logistical challenge to conducting home visits was the child not being at home, most often due to school. This was mentioned more frequently by HCWs than CHWs.

*“We were sometimes told that the child is in class and we would have to come back after or the next day or in the evening…If she tells you to come back in the evening because the child went to school, that’s when you will come back. If it’s the next day, you’ll come back the next day depending on her schedule and the availability of the children.” (CHW).*

They were also told the child was at church or staying with another relative, or the parents were working away from home. A number of providers felt that in some of these cases, the parents had no intention of testing their children.

*“You can talk to a woman who is hiding her own biological children. She sometimes tells you that the children aren’t there, they’ve traveled…we tell them that it’s for their own good and that of their children. We tell them but they still keep hiding their children.” (CHW).*

The most significant obstacle raised by HCWs and CHWs to index/contact attendance at the health facility was the cost of travel, particularly for those traveling long distances from their homes. Index clients may live far away from their chosen facility and avoid clinics closer to their communities out of fear of inadvertent disclosure and/or stigmatization by their neighbors. Cost of travel to the clinic was exacerbated when the caregiver had multiple children to be tested.

*“…A mother who brings two or three children to you, in her mind when we call them, …we also must reassure them that the [reimbursement of] transportation is there. If the transportation is not there…it is going to be a hindrance, we are really going to be stuck!” (HCW).*

Respondents also described transport reimbursement or other payment for activities under the PACE initiative to HCW or CHW as insufficient. This included transport to households for HIV testing for clients who could not travel to the facility and insufficient telephone funds to call index clients.

### Effect of COVID-19 pandemic

Respondents reported a perceived decrease in client acceptance of home visits and general facility attendance prompted by the COVID-19 pandemic. The primary reason cited for the decrease in attendance at health facilities was client fear of exposure to the severe acute respiratory syndrome coronavirus 2 (or SARS-CoV-2), often resulting from circulating rumors or news of positive cases. Several HCWs and CHWs also said clients were fearful of being tested and potentially quarantined if they presented at a health facility, which contributed to decreased attendance.

*“At the time of the COVID-19 pandemic, the bad consequence was a reduction in patient attendance. Many were afraid to arrive at the facility even on the day of their appointment.” (CHW).*

Furthermore, a few respondents mentioned that transportation limitations and lockdown restrictions contributed to the decline in facility attendance. A few others added the support group meetings were suspended, losing another opportunity to engage with the health system. In contrast, two HCWs said COVID-19 had not impacted their facility turnout, with one explaining that one community had denied the pandemic’s existence. The other said clients were hesitant to come in, but still showed up and were reassured after talking with providers.

According to several respondents, community outreach was temporarily suspended for a time during the pandemic. When home visits were part of service delivery, providers shared a mix of experiences – some clients accepted them, particularly if their drugs were delivered as well, while others were hesitant or refused home visits due to fear of exposure.

*“When we went to see some of them, we found “no visits“ posted and we had to go back home. When we called them on the phone, they asked if we didn’t know what was going on.” (CHW).*

Following safety precautions and the provision of personal protective equipment somewhat mitigated providers’ own fears of exposure while conducting home visits. However, half of HCW and CHW reported inadequate protection and several (primarily HCWs) were reluctant to visit communities.

*“The protective materials that we have now, if we had had that before, the activities would have gone on as usual, but we did not have enough masks; we often used fabric masks. Afterwards they had to be washed. It was difficult to find medical masks.” (HCW).*

### Provider strategies to address gaps

HCWs and CHWs offered strategies they have implemented to address challenges encountered along the pediatric index testing cascade. To ensure elicitation was completed for all clients, participants described repeating the elicitation process with those who say they do not have children; eliciting names of children before giving the index case their positive HIV result; and checking if a list was completed at the time of VL sample collection.

*“To make sure that all children have been tested, especially those who have reported having children…we need to see first in our records if all children are tested and then continue to ask if there are other children to be tested.” (HCW).*

To address logistical tracing challenges at household level, both HCWs and CHWs recommended arranging home visits around school schedules, encouraging mothers to bring children for HIV testing at their next drug refill visit, and employing creative tactics to obtain accurate home addresses. Strategies to bring children to health facilities largely centered around incentivizing families through the offer of food at the clinic or transport reimbursement.

*“The most important thing for us is that the patient arrives at the health center with his or her children, that we test them and then we find 2,000 or 3,000 CDF [Congolese Franc] to help him or her return home. That is what we find difficult.” (HCW).*

The most frequently mentioned strategies involved encouraging index cases of sero-discordant couples to test their children, even if they did not want to disclose their status to their partner, or have their children tested for HIV without one partner becoming aware. This included offering HIV testing under the guise of a broader community or door-to-door campaign or implementing the ‘one by two’ approach. The latter strategy involves conducting a home visit at the index household and another home nearby to give the impression that the client’s family is not being targeted for testing in an effort to minimize stigmatization.

*“Nevertheless, for all these cases, we go down anyway, as we do the ‘one by two’ to mask. We start with the plot before them, so that if her husband is there, he will not be frustrated because we have started doing this [HIV test] with the other neighbors, and now it is his children’s turn.” (HCW).*

Other suggestions included arranging a household visit or travel to the facility when the husband is not at home, offering to go to a private place outside the home or a family confidant’s, or disguising the HIV test as a test for malaria or yellow fever. One CHW said,

*“…the best thing for us is to go down to the field to meet these children and their mothers when their fathers are not there to accompany them to the facility and test them.”*

One HCW responded that they always encourage clients to disclose their HIV status. More generally, some HCWs talked about the extensive counseling they do over the course of many visits until parents agree to test their children. Another HCW summed up their efforts to find eligible children and ensure testing.

*“At every visit, or every occasion that we meet index cases and we could discuss, we always try to find strategies to reach these children. And it is because of this that some children are finally tested, even if the others are not, and we continue to offer testing of children.” (HCW).*

## Discussion

Following intensified pediatric case finding, we found a high HIV positivity rate of 6.7%, with 81% of CLHIV ≥5 years; nearly all children were linked to treatment. At the start of the intensified effort, less than half of ART clients had their contacts elicited. After nearly a year and a half, all new and existing ART clients had undergone elicitation for their biological children < 15 years who were undiagnosed with HIV. Gaps in the index testing cascade persisted over time, with only a minimal reduction in unsuccessful tracing for HIV testing during the first month of the initiative and throughout the period (17.4% vs. 16.7%). Community and health workers attributed these gaps primarily to child HIV testing refusals due to non-disclosure among parents and logistical and financial means of transporting families to facilities, or providers to communities, for HIV testing. During some of this period, movement restrictions and individual fears of exposure related to the COVID-19 pandemic may have also played a role in less optimized implementation of the approach.

Other studies have similarly found large numbers of undiagnosed children of people living with HIV, particularly older children who may have more limited interaction with the health system than their younger counterparts. This raises the need to focus on school-aged children who are not often targeted by health programs. Evidence from other countries, such as South Africa and Malawi, revealed challenges with elicitation and testing of biological children, with almost half of clients living with HIV enrolled in treatment services had untested household members [5, 8] and up to 81% of children of adult ART clients were reportedly not tested for HIV. Older children 9–14 years were the least likely to be tested compared to younger children [4, 5].

Our study found a higher HIV positivity yield when compared to children of similar ages in Côte d’Ivoire (5% seropositivity rate) [9] and Kenya (4.5%) [10]. Although varying from one country to another, index testing in children consistently demonstrates a high seropositivity compared to other entry points for pediatric HIV testing. For example, HIV positivity yield in outpatient and inpatient departments was 1.2 and 1.6% respectively, compared to 4.5% with family testing [10]. This suggests that index testing is a promising strategy for identifying CLHIV, particularly in countries with low coverage with HIV services [8]. In addition to index testing gaps that may have led to delayed diagnoses of HIV in children, the high positivity rate in our study could also be attributable in part to other service-related missed opportunities as DRC has one of the lowest rates in the region of PMTCT program coverage at 61% [2].

Pediatric case finding can be optimized by integrating index testing into multiple facility entry points or combining facility and community approaches. In Côte d’Ivoire, the overall HIV seropositivity represented 5% among children [9]. In Cameroon, high rates of HIV infection were found among siblings/descendants of HIV-positive index clients (22.2%), TB treatment unit attendees (11.4%) and hospitalized children/adolescents (5.6%) [11] Similar results were obtained in Nigeria in which the odds of identifying a child living with HIV were significantly higher in specialized pediatric units and services, including the TB clinic, pediatric inpatient ward, and family index testing, when compared to general outpatient care [12]. The introduction of community-based strategies in addition to facility-based testing can be critical to closing the gap and finding CLHIV. This is especially true for children over 2 years of age who have limited touchpoints with the formal healthcare system [13].

Despite the high HIV-positivity yield, we found about 17% of children were not reached for HIV testing. Qualitative data from providers suggests one reason for this is fear of HIV status disclosure among index female clients. Their fear of negative consequences for having their children tested may result in inadvertent disclosure of maternal status, which can lead to intimate partner violence or other harms for the mother and her child [5]. In contrast, findings from a Kenya study identified barriers to parental disclosure that centered primarily around child-focused consequences, including concerns of child distress, stigmatization from their children or subsequent inadvertent disclosure of their status by their children to others [14]. While continued counseling was offered as a strategy by a few providers in our study, short-term solutions were also described to arrange for HIV testing, with the consent of the index parent, but without the other parent’s knowledge. Health worker-supported family HIV disclosure, both for previous and current sexual relationships is one strategy that may help to facilitate subsequent HIV testing for undiagnosed children to address this issue at the root level [15].

Documented multi-level barriers to pediatric HTS delivery and uptake include fear of a positive test, an inaccurate perception of risk, challenges with paternal consent, school schedules and logistical barriers, including the perceived costs of testing and care [16–18]. In our study, providers described financial and logistical barriers experienced by caregivers to travel to the health facility for child testing and by HCWs and CHWs to deliver community-based testing. These barriers may be exacerbated for caregivers of multiple children with an unknown HIV status [19]. The mobility of testing teams may also be limited, especially when households are difficult to reach or located a far distance from facilities. To overcome these challenges, many HCWs and CHWs suggested that programs incorporate sufficient costs to cover both transportation and communication needs.

The COVID-19 pandemic threatened to undermine progress in eliciting and testing children by exacerbating existing barriers to HIV testing. There were fewer opportunities for elicitation and testing in facility and community settings, at least temporarily during the COVID-19 pandemic, though it is difficult to estimate the extent to which this had lasting influence on pediatric HTS. The COVID-19 pandemic exposed health systems’ weaknesses and may have stymied progress, resulting in a potential increase in the number of new pediatric infections due to COVID-19-related care disruptions [20]. The pandemic and the response to it exacerbated existing disparities and access to HIV prevention services that may have sustained effects over time. The COVID-19 pandemic posed significant challenges to HIV prevention that go beyond changes due to quarantine and community containment measures [20].

This study has some noted limitations. The quantitative data were based on routine documentation at site level, which may contain irregularities or inaccuracies. Also, it was not possible to separate out reasons for ineligibility among the 11% of excluded female ART clients; some may have had children < 15 years with a known HIV status, while others may have been excluded because their children were ≥ 15 years. Similarly, we could not disentangle the number of children not reached for testing from those whose parents declined testing. For the qualitative component, there may have been selection bias, which could have underestimated the gaps or inflated provider-imposed strategies. The providers recruited for the study were predetermined as those trained under the PACE initiative. Those selected to participate in the training by program managers were most involved in HIV services and most likely to be receptive to training. Although as data collection was qualitative, the purpose was not to be generalizable. We also only captured perspectives of providers, not clients themselves, who have their own unique experiences and views. However, the providers represented the broader perspective based on their interactions with beneficiaries and are the most appropriate voices to describe ‘supply side issues’, such as transportation and the emphasis on elicitation. We also included CHWs, who not only play a key role in index testing activities, but they are members of the same communities as clients and beneficiaries of the same health services.

## Conclusions

We found a high HIV positivity yield among previously undiagnosed children following the introduction of strategies at facility, community and health zone level to improve pediatric case identification, integrated into existing HIV testing services. In an effort to understand the gaps along the service continuum, health and community workers identified financial and logistical barriers to reaching children for testing and non-disclosure among parents as an obstacle to testing acceptance for their children. Ensuring adequate resources for communication, transportation, and protective equipment as needed and supporting disclosure among couples, while continuing to emphasize elicitation of biological children of ART clients can help to optimize pediatric case finding. This ensures children, particularly older children with limited health system interaction, can be diagnosed and receive life-saving treatment.

### Supplementary Information

**Additional file 1.**

## Data Availability

All quantitative data generated or analyzed during this study are included in this published article. While we do not have permission to share qualitative transcripts in full per the terms of study consent, relevant coded segments of interview/focus group data are available from the corresponding author on reasonable request.

## Abbreviations

ART: Antiretroviral treatment
CHW: Community Health Worker
CLHIV: Children living with HIV
EGPAF: Elizabeth Glaser Pediatric AIDS Foundation
FGD: Focus group discussion
HCW: Healthcare Worker
HIV: Human Immunodeficiency Virus
HTS: HIV Testing Services
HZ: Health Zone
IDI: In-depth individual interview
PACE: Pediatric-focused accelerated case finding effort
