# Supplementary material for: Optimizing HIV case identification among children and understanding remaining gaps in pediatric HIV testing in Kinshasa, DRC
Source: BMC Pediatr. 2024 Jan 4;24:10. doi: 10.1186/s12887-023-04485-1 (PMC10765655; doi:10.1186/s12887-023-04485-1)
Supplement: Supplementary file 1 — Additional file 1. [file 12887_2023_4485_MOESM1_ESM.docx]

**Additional file 1. Pediatric index testing flow diagram at baseline, March 2020**

Female index clients ≥18 years on ART identified

N=4,737

Clients with ≥1 biological child <15 years with unknown HIV status

N=3,337

Clients with contacts documented in files who did not meet criteria

N=1,400

Clients who underwent child contact elicitation

N=1,634

Contacts tested for HIV by age bands:

- Contacts 0 – 4 years (N=316)
- Contacts 5 – 9 years (N=810)
- Contacts 10 – 14 years (N=793)

Total: 1,919

Clients not reached for contact elicitation

N=1,703

Listed contacts eligible for HIV testing by age bands:

- Contacts 0 – 4 years (N=393)
- Contacts 5 – 9 years (N=961)
- Contacts 10 – 14 years (N=973)

Total: 2,327

Contacts not tested:

- Contacts 0 – 4 years (N=77)
- Contacts 5 – 9 years (N=151)
- Contacts 10 – 14 years (N=180)

Total: 408

Contacts tested HIV positive by age bands:

- Contacts 0 – 4 years (N=24)
- Contacts 5 – 9 years (N=47)
- Contacts 10 – 14 years (N=41)

Total: 112

All contacts diagnosed as HIV positive were linked to treatment (N=112)
